# Long-Term Remission with Novel Combined Immune-Targeted Treatment for Histiocytic Sarcoma Accompanied by Follicular Lymphoma: Case Report and Literature Review

**DOI:** 10.3390/ijms25137293

**Published:** 2024-07-02

**Authors:** Minyue Zhang, Fei Xiao, Jianchen Fang, Zebing Liu, Yanying Shen, Di Zhu, Yiwei Zhang, Jian Hou, Honghui Huang

**Affiliations:** 1 Department of Hematology, Ren Ji Hospital, School of Medicine, Shanghai Jiao Tong University, Shanghai 200127, China; zhangminyue@renji.com (M.Z.); xiaofei@renji.com (F.X.); zhudi@renji.com (D.Z.); zhangyiwei@renji.com (Y.Z.); 2 Department of Pathology, Ren Ji Hospital, School of Medicine, Shanghai Jiao Tong University, Shanghai 200127, China; fangjianchen@renji.com (J.F.); liuzebing@renji.com (Z.L.); shenyanying@renji.com (Y.S.)

**Keywords:** histiocytic sarcoma, follicular lymphoma, immune-targeted therapy, Daratumumab, Pazopanib, Tislelizumab

## Abstract

Histiocytic sarcoma (HS) is an extremely rare but aggressive hematopoietic malignancy, and the prognosis has been reported to be rather unfavorable with a median overall survival of merely 6 months. We presented a 58-year-old female patient complaining of abdominal pain and fever, who was admitted to our institution in September 2021. Fluorine-18-fluorodeoxyglucose (FDG) positron emission tomography–computed tomography (PET/CT) scan showed enlargement of generalized multiple lymph nodes. Subsequently, laparoscopic retroperitoneal lesion biopsy and bone marrow aspiration were performed. The pathological findings indicated the diagnosis of HS concurrent with follicular lymphoma. The immunohistochemistry (IHC) staining of the tumor lesion revealed a high expression of CD38 and PD-L1 proteins. Furthermore, *KRAS* gene mutation was identified by means of next-generation sequencing. The patient exhibited poor treatment response to both first- and second-line cytotoxic chemotherapies. Therefore, she underwent six cycles of Daratumumab (anti-CD38 monoclonal antibody), Pazopanib (multi-target receptor tyrosine kinases inhibitor) combined with third-line chemotherapy, followed by involved-site radiotherapy and maintenance therapy with the PD-1 inhibitor Tislelizumab. Long-term partial remission was finally achieved after multi-modality treatment. Duration of remission and overall survival reached 22 and 32 months, respectively. Our case indicated that immuno-targeted treatment coupled with chemotherapy and radiotherapy might constitute a potential therapeutic option for HS.

## 1. Introduction

Histiocytic sarcoma (HS) is an extremely rare lymphohematopoietic tumor. The tumor cells derive from the monocyte–macrophage lineage and are highly aggressive [1]. It is categorized as an independent disease under the broad of histiocytic and dendritic cell tumors in the fifth edition of the *WHO Classification of Tumors of Hematopoietic and Lymphoid Tissues* [1]. The etiology of HS remains unknown, and it is rare in clinical practice. As such, the diagnosis and differential diagnosis of HS can be challenging, and it is frequently misdiagnosed as other malignant tumors. A small number of patients with HS were also accompanied by lymphoma [2,3,4]. Patients with HS had poor prognoses due to lack of effective treatments [5]. Herein, we presented a case of HS concurrent with follicular lymphoma achieving objective disease response and favorable prognosis with a combination of frontline immune-targeted therapy.

## 2. Case Presentation

**Figure 1:**
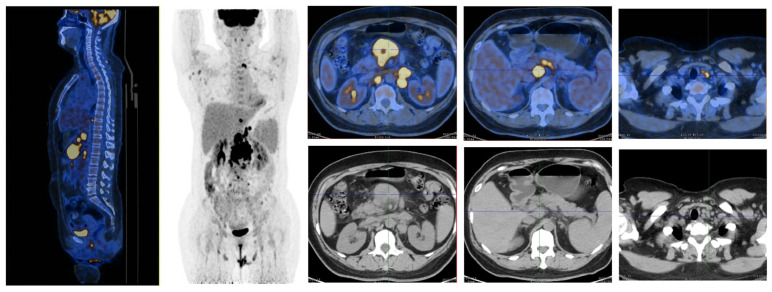
The positron emission tomography–computed tomography (PET/CT) images demonstrated extensive enlargement of the lymph nodes with increased fluorine-18-fluorodeoxyglucose (FDG) metabolism at initial diagnosis.

**Figure 2:**
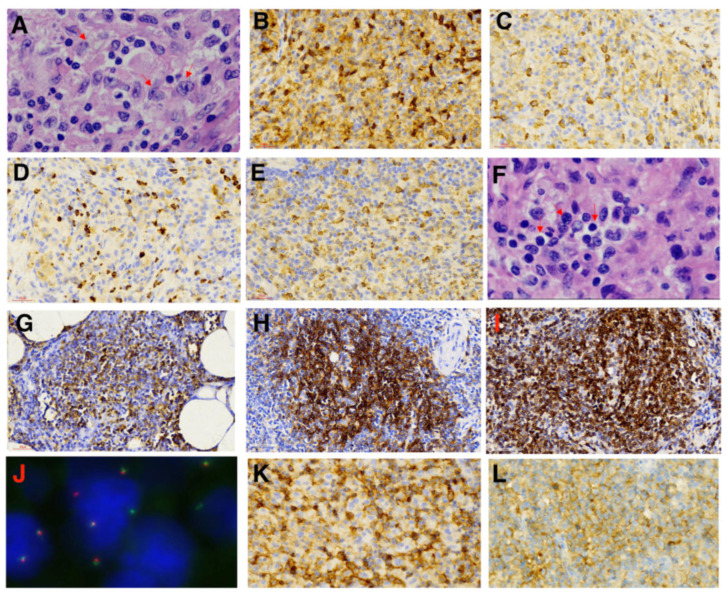
Pathological analysis of retroperitoneal lesion. Images of H&E staining (100× oil) of histiocytic sarcoma cells (**A**) and immunohistochemistry (IHC) staining (400×) of CD163 (**B**), lysosome (**C**), CD4 (**D**), CD68 (**E**), PD-L1 (**K**), and CD38 (**L**); Images of H&E staining (100× oil) of follicular lymphoma cells (**F**), IHC staining (400×) of CD20 (**G**), CD10 (**H**), and BCL-2 (**I**), as well as BCL2 gene translocation (**J**) by fluorescence in situ hybridization (FISH). The red arrows in Figure 2A and Figure 2F represented histiocytic sarcoma cells and follicular lymphoma cells, respectively.

**Figure 3:**
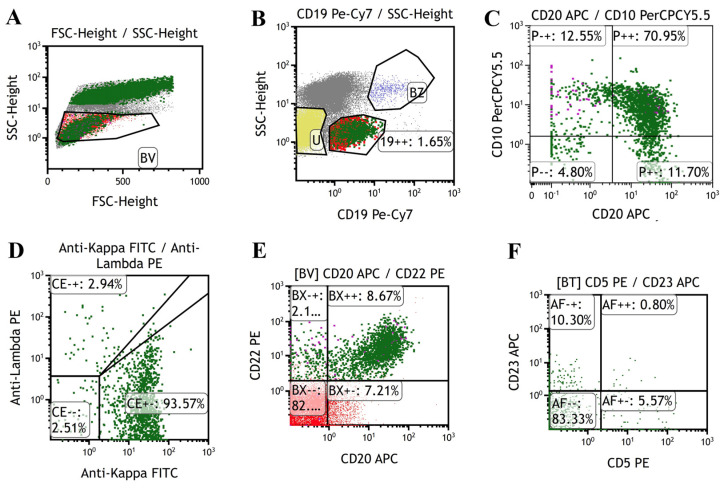
Representative images of multiparameter flow cytometry analysis of bone marrow at initial diagnosis indicating the involvement of bone marrow by follicular lymphoma cells. The tumor cells (green dots circled) were small in size (**A**) with positive for CD 19 (**B**), CD 20 (**C**), CD10 (**C**), and CD22 (**E**) while negative for CD5 or CD23 (**F**). The tumor cell had a restrictive expression of membranous immunoglobulin light chain Kappa (**D**).

A 58-year-old female patient presented with asymptomatic retroperitoneal lymphadenectasis by ultrasonography during her physical examination one year before admission. In September 2021, due to upper abdominal pain and recurrent fever with a maximal body temperature of 38 °C for two weeks, she was admitted to our department. Physical examination showed that a round mass (about 3*3cm in size) which was hard and slightly tender was palpated below the xiphoid process. Fluorine-18-fluorodeoxyglucose (^18^FDG) positron emission tomography–computed tomography (PET/CT) scan was performed and demonstrated generalized enlargement of the lymph nodes with increased FDG metabolism (SUVmax = 5.6–25.8) in the supraclavicular region, axillary region, celiac region, and the inguinal area (Figure 1). The largest one was found in the mesenteric area under the body of the pancreas, approximately 4 × 4 cm in size. Then the patient underwent laparoscopic resection of the retroperitoneal lesion. Postoperative pathology indicated histiocytic sarcoma based on Hematoxylin and Eosin (H&E) staining (Figure 2A) and immunohistochemistry (IHC) staining, revealing that the tumor cells were positive for CD163, Lysosome, CD4, and CD68 (Figure 2B–E), whereas they were negative for myeloid (MPO), T cell (CD3 and CD8), B cell (CD19), and dendritic cell (CD1a and CD21) lineage markers. The Ki-67 proliferation index was 70%. Additionally, follicular lymphoma (FL) was diagnosed, as detected by small number of small lymphocytes diffusely infiltrating surrounding adipose tissue (Figure 2F), which were positive for CD20 (Figure 2G), CD10 (Figure 2H), and BCL-2 (Figure 2I) by IHC staining and were associated with BCL2 gene-related translocation t(18q21) by fluorescence in situ hybridization (FISH) (Figure 2J). Moreover, the bone marrow examination also suggested the infiltration of BM by FL, supported by the abnormal small lymphocytes expressing CD19, CD20, CD22, and CD10 and a restrictive expression of membranous immunoglobulin light chain Kappa by flow cytometry (Figure 3). The patient was ultimately diagnosed with HS accompanied by FL.

**Figure 4:**
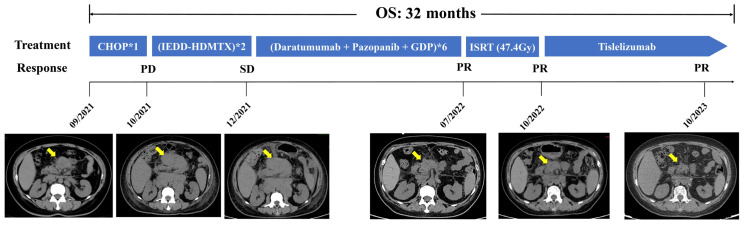
Representative computed tomography (CT) images, treatment, and clinical courses of this patient. CHOP, cyclophosphamide, vincristine, doxorubicin, and prednisone; GDP, gemcitabine, cisplatin, and dexamethasone; IEDD-HDMTX, ifosfamide, etoposide, liposomal doxorubicin, dexamethasone, and high-dose methotrexate; PD, progressive disease; PR, partial remission; SD, stable disease. The yellow arrow indicated enlarged the lymph nodes in mesenteric area.

The patient started to receive cytotoxic chemotherapy in September 2021, including one cycle of the CHOP regimen (cyclophosphamide, vincristine, doxorubicin, and prednisone) and two cycles of the IEDD-HDMTX regimen (ifosfamide, etoposide, liposomal doxorubicin, dexamethasone, and high-dose methotrexate). However, lymphadenopathy had no improvement in CT scans after chemotherapy. To explore potential therapeutic targets, further IHC staining was performed and demonstrated that the tumor cells were positive for PD-L1 (Figure 2K) and CD38 (Figure 2L). Moreover, the tumor biopsy specimen was sequenced by capture-based next-generation DNA/mRNA sequencing with a panel containing 495 hematological malignancy-related genes (Geneseeq Technology Inc., Toronto, ON, Canada). In addition to the *BCL2-IgH* fusion gene, a total of 22 somatic mutations were identified, including *KRAS* (c.183A > C, p.Q61H) and *KMT2D* (c.12374del, p. S4125Lfs*17) mutations (Supplementary Table S1). Thereafter, a combination of immune-targeted therapy with cytotoxic chemotherapy consisting of anti-CD38 monoclonal antibody Daratumumab, multi-target receptor tyrosine kinases inhibitor Pazopanib, plus the GDP regimen (gemcitabine, cisplatin, and dexamethasone) was administered in December 2021. Following six cycles of treatment, the CT scan showed that the sum of the perpendicular diameters (SPDs) of the enlarged lymph nodes was reduced by about 50% in comparison to that before treatment (Figure 4), indicating partial remission (PR). Subsequently, the patient received involved-site radiotherapy (ISRT) to the left side of the neck and abdomen at a total dose of 47.4Gy, followed by PD-1 inhibitor Tislelizumab maintenance therapy every 3–4 weeks. An efficacy evaluation with CT scan imaging demonstrated that the SPD of the enlarged lymph nodes was decreased by approximately 70% (Figure 4). The patient remains alive up to now; the duration of remission (DOR) and overall survival (OS) reached 22 and 32 months, respectively.

## 3. Discussion

HS is an aggressive and rare hematopoietic malignancy, which is derived from the monocyte–macrophage system. We reported a Chinese female patient with HS and co-occurring FL was successfully treated with frontline combined immune-targeted therapy. A series of interesting observations were obtained from this case.

Currently, there have been few large-scale studies on HS until now. Most have been clinical case reports from different research centers [2,3,4,6,7,8,9,10,11]. Kommalapati et al. retrospectively analyzed the clinical features of 159 patients diagnosed with HS in the Surveillance, Epidemiology, and End Results (SEER) database from 2000 to 2014 [5]. The age-adjusted incidence rate of HS was 0.17/1,000,000 individuals. The median age of onset for HS was 63 years old. The most common sites of tumor involvement were skin and connective tissue (35.8%), followed by the lymph nodes (17%). Furthermore, a small minority of patients with HS were reported to co-exist with or be secondary to lymphoid neoplasms, which are summarized in Supplementary Table S2 [2,3,4,10,12,13,14,15,16,17,18,19,20,21,22,23,24,25,26]. Some studies indicated the transdifferentiation of HS from lymphoid neoplasms, as evidenced by a clonal relationship between these two diseases through identical clonal *IG* gene rearrangements or an *IGH/BCL2* sequence. In the current case, although the patient had a one-year history of an asymptomatic enlarged retroperitoneal lymph node before admission, no convincing evidence showed a common clonal origin between HS and FL. Therefore, it is difficult to infer whether HS was transdifferentiated from FL for the current patient.

The diagnosis primarily depends on histopathology with H&E staining and IHC staining [27,28]. IHC of HS suggested that the tumor cells expressed at least two mature histiocytic markers, including CD68, CD163, CD4, and Lysozyme [27,28]. Additionally, there were reports in the literature of tumor cells over-expressing PD-L1 [29,30,31,32] and CD38 [33], which is consistent with our patient and regarded as the potential therapeutic targets. Recently, genetic alterations of HS have been investigated in a series of reports [10,26,27,28,34,35,36,37]. Egan C, et al. identified genetic alterations within the RAS/RAF/MAPK pathway by whole-exome sequencing and RNA sequencing in HS patients, including *NF1*, *MAP2K1*, *PTPN11*, *BRAF*, *KRAS*, *NRAS*, and *LZTR1* [36]. In addition, other studies reported that patients with HS harbored a recurrent *KMT2D* gene mutation [10,26,37], which is an epigenetic regulator. In the current study, this patient was also found to have mutations in the *KMT2D* and *KRAS* genes by NGS. Overall, these molecular features of HS indicated that alterations in the RAS/RAF/MAPK pathway and chromatin regulation may be considered as future therapeutic targets.

To date, there is no standard treatment regimen for HS. Lymphoma-type chemotherapy protocols, such as CHOP, ICE, and bendamustine, have been widely used in clinical practice [18,38,39,40]. Application of allogeneic or autologous hematopoietic stem cell transplantation in relapsed or refractory disease was also reported in a series of HS cases [40,41]. However, the outcomes were inconsistent. Furthermore, with in-depth study on the molecular features of HS, some novel targeted drugs have been increasingly applied in the treatment of refractory recurrent HS, with a kinase inhibitor (imatinib and sorafenib), anti-vascular endothelial growth factor (VEGF) antibodies (bevacizumab), an MEK inhibitor (trametinib), a BRAF inhibitor (vemurafenib), and immune checkpoint inhibitors (PD-1 inhibitors), leading to favorable clinical responses [30,32,42,43,44,45,46,47]. In this case, combined immune-targeted therapies were implemented based on the results of genetic molecular testing of tumor tissues. Daratumumab, a humanized, IgG1-κ-type anti-CD38 monoclonal antibody, has been approved for the treatment of multiple myeloma. Pazopanib is a multi-target tyrosine kinase inhibitor, which can suppress the RAS/RAF/MAPK signaling pathway [48,49]. It has been widely used in the treatment of renal carcinoma [48,49]. As CD38 and PD-L1 were highly expressed by IHC staining and a *KRAS* mutation was detected by NGS in this patient, multi-modality therapy with Daratumumab, Tislelizumab, and Pazopanib was successively implemented and achieved a favorable response. To the best of our knowledge, this is the first report of Pazopanib and Daratumumab being applied in the treatment of HS.

HS is a highly aggressive malignant hematological tumor with poor prognosis [5]. The median OS of patients with HS was reported to be approximately 6 months from the National Cancer Database (NCDB) [50]. Our patient was still alive and maintained disease remission with an OS of 32 months after receiving immune-targeted treatment combined with chemotherapy and radiotherapy, which was far beyond that of HS patients registered in the NCDB.

## 4. Conclusions

HS is an extremely rare hematological malignancy with poor prognosis. We presented the first case report of HS co-existing with FL benefitting from novel combined immune-targeted therapies and obtaining favorable therapeutic effects. We recognize the clinical importance of genomic and molecular studies for the establishment of individualized treatment in such patients with an otherwise dismal outcome.

## Data Availability

The original contributions presented in this study are included in this article/Supplementary Materials. Further inquiries can be directed to the corresponding author.

## Supplementary Materials

The supporting information can be downloaded at: https://www.mdpi.com/article/10.3390/ijms25137293/s1.
